# Focus on Peripheral Biomarkers of Mental Disorders

**DOI:** 10.3390/brainsci12060756

**Published:** 2022-06-08

**Authors:** Francesco Bartoli, Giuseppe Carrà

**Affiliations:** 1 Department of Medicine and Surgery, University of Milano-Bicocca, Via Cadore 48, 20900 Monza, Italy; giuseppe.carra@unimib.it; 2 Division of Psychiatry, University College London, 6th Floor, Maple House, 149 Tottenham Court Road, London W1T 7NF, UK

Personalized approaches in psychiatry, albeit being extensively explored in the literature since the early 2010s [1,2,3,4], are far from being routinely used in clinical practice. Indeed, compared with other clinical fields, which have already filled the translational gap, psychiatry has not yet benefited from the advanced methodologies needed for precision medicine [1]. Although research resources have been extensively allocated to the aims of this approach [5,6], the biological underpinnings of mental disorders remain unknown [7]. The identification of valid biomarkers would represent an important step towards precision psychiatry with the goal of supporting personalized approaches for prevention, diagnosis, and treatment of mental disorders in clinical practice [8]. Consistently, in the last few years, research has paid attention to this topic, generating several systematic syntheses on biomarkers belonging to inflammatory, immune, and oxidative stress pathways [8,9,10,11,12,13,14,15,16,17]. Nonetheless, evidence emerging from this large body of scientific literature around different mental disorders is still not convincing and requires additional studies [8].

Following this perspective, the goal of this special issue entitled “*Peripheral Biomarkers of Mental Disorders and Related Clinical Features*” was to provide additional insight into this topic, including 10 relevant studies from key experts in the field [18,19,20,21,22,23,24,25,26,27], with the majority of them investigating the cutting-edge topics of affective disorders.

In our opinion paper [18], co-authored with the pioneer of purinergic signaling, Professor Geoffrey Burnstock [28,29], we explored the neurobiological background of the hypothesized link between the purinergic pathway and mood disorders. The potential role of the adenosine and ATP-mediated signaling at P1 and P2 receptors in depression, the antidepressive effects of non-selective adenosine antagonists, and the promising role of peripheral adenosine metabolites as biomarkers of depression were all extensively described.

Fusar-Poli et al. [21], testing the role of inflammation in different phases of bipolar disorder, investigated whether neutrophil-to-lymphocyte (NLR), platelet-to-lymphocyte (PLR), and monocyte-to-lymphocyte (MLR) ratios might represent potential biomarkers of mood episodes in 294 individuals with bipolar disorder. NLR, PLR, and MLR in study participants with hypo/manic episodes were significantly higher than in those with a depressive episode, and PLR was estimated as an independent predictor of mania. In another study, exploring the role of parathyroid hormone (PTH), vitamin D, and serum calcium in 199 individuals with bipolar disorder, Steardo et al. [25] found that PTH levels correlated with different clinical characteristics, such as psychotic features, suicidal behaviors, and the number of mood relapses. The authors suggested that calcium homeostasis could play a role in bipolar disorder and that PTH levels might be correlated with the clinical severity of the disorder. Moreover, Reginia et al. [23], comparing 30 individuals suffering from bipolar disorder and 30 healthy controls, hypothesized abnormalities in regenerative processes in bipolar disorder, even though no differences in stem-cell levels between groups were estimated. In addition, in their opinion article, Wollenhaupt-Aguiar et al. [25] overviewed putative molecular pathways and biomarkers of neuroprogression possibly affecting clinical outcomes, cognition, and functioning of bipolar disorder; they suggested that multiple factors play a role in bipolar disorder, factors such as inflammation, oxidative stress, impaired calcium signaling, mitochondrial dysfunctions, and impaired neuroplasticity.

Berardelli et al. [19], in a systematic review of data from 36 studies, suggested an involvement of the hypothalamic–pituitary–adrenal (HPA) axis on the pathophysiological processes associated with suicidal behaviors, discussing the potential implications in terms of relevant treatments.

Vismara et al. [26] also ran a review, this on the available evidence about the peripheral biomarkers of anxiety disorders, showing mixed findings for the cerebrospinal fluid and blood biomarkers related to neurotransmitters, neuropeptides, the HPA axis, neurotrophic factors, and the inflammatory system.

Finally, Maes et al. [22], using data from 80 participants with schizophrenia and 40 healthy controls, and integrating clinical information—such as specific symptoms of psychosis, self-reported quality of life, memory, and executive functions—with different inflammatory, immunological, and oxidative stress biomarkers, hypothesized new diagnostic subclasses of schizophrenia.

The content of this special issue may represent a preliminary, though still meaningful, contribution to the scientific evidence on biomarkers of mental disorders and related behaviors. Main efforts of research in biological psychiatry are moving towards novel approaches and new advancements for diagnosis and treatment of mental disorders. Relevant progress in the identification of peripheral biomarkers may improve the effectiveness of mental health care and address some of the unmet needs in the clinical management of severe mental disorders.

This special issue is also our tribute to Professor Geoffrey Burnstock, who died a few months after the publication of our opinion article at the age of 91 [28,29]. His contribution to our paper was generous and enthusiastic. Throughout the world there are many who owe him a similar debt of gratitude.
